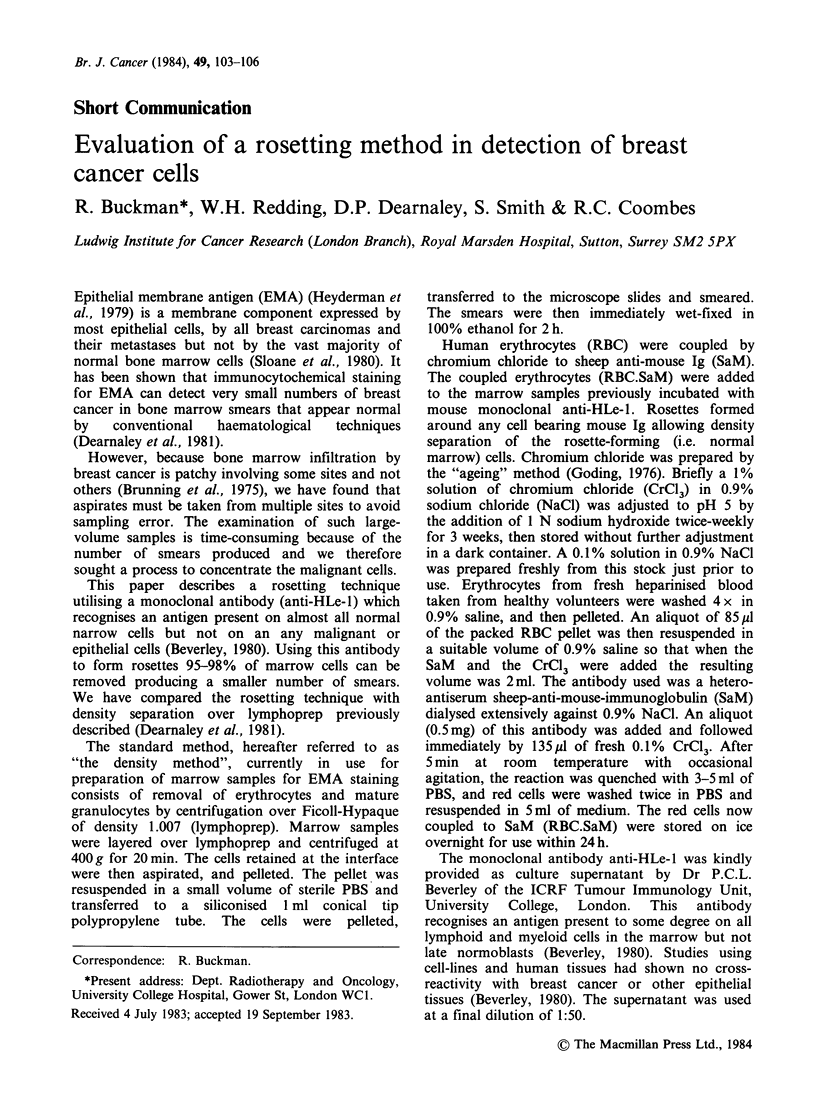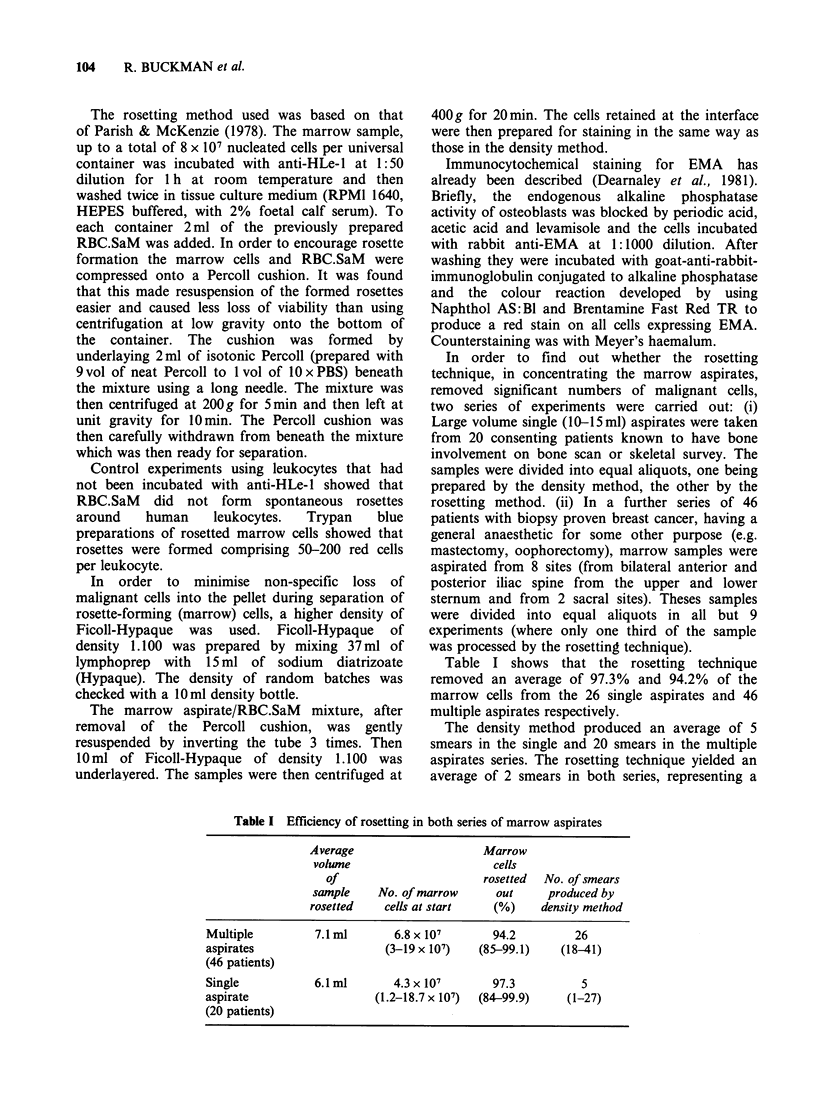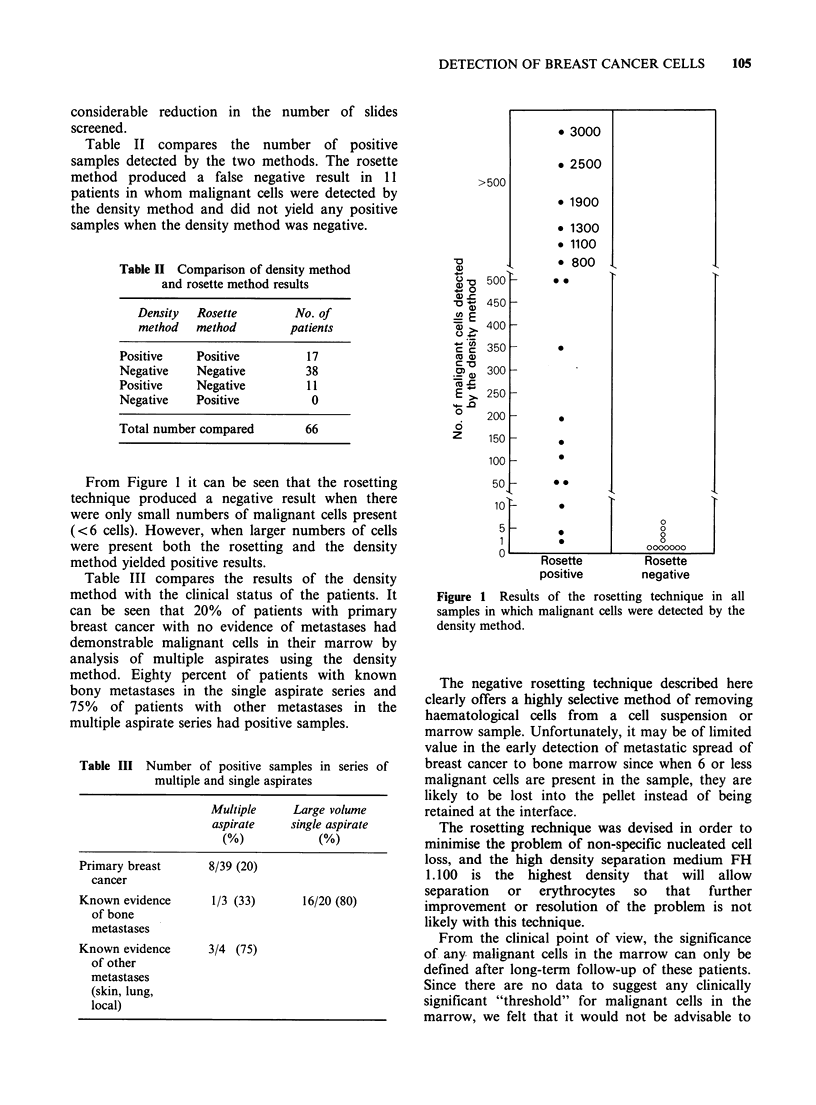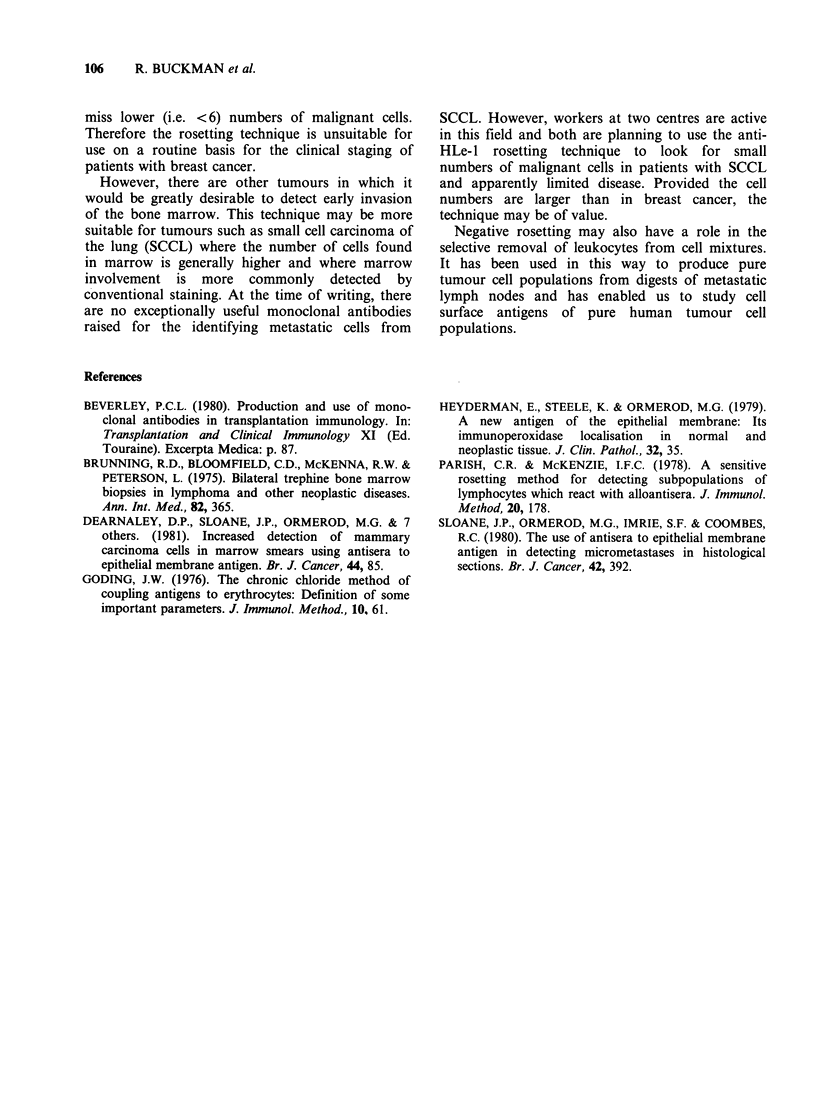# Evaluation of a rosetting method in detection of breast cancer cells.

**DOI:** 10.1038/bjc.1984.16

**Published:** 1984-01

**Authors:** R. Buckman, W. H. Redding, D. P. Dearnaley, S. Smith, R. C. Coombes


*Br. J. Cancer* (1984), **49**, 103–106

**Short Communication**

### Evaluation of a rosetting method in detection of breast cancer cells

R. Buckman*, W.H. Redding, D.P. Dearnaley, S. Smith & R.C. Coombes

*Ludwig Institute for Cancer Research (London Branch), Royal Marsden Hospital, Sutton, Surrey SM2 5PX*

Epithelial membrane antigen (EMA) (Heyderman *et al.*, 1979) is a membrane component expressed by most epithelial cells, by all breast carcinomas and their metastases but not by the vast majority of normal bone marrow cells (Sloane *et al.*, 1980). It has been shown that immunocytochemical staining for EMA can detect very small numbers of breast cancer in bone marrow smears that appear normal by conventional haematological techniques (Dearnaley *et al.*, 1981).

However, because bone marrow infiltration by breast cancer is patchy involving some sites and not others (Brunning *et al.*, 1975), we have found that aspirates must be taken from multiple sites to avoid sampling error. The examination of such large-volume samples is time-consuming because of the number of smears produced and we therefore sought a process to concentrate the malignant cells.

This paper describes a rosetting technique utilising a monoclonal antibody (anti-HLe-1) which recognises an antigen present on almost all normal narrow cells but not on an any malignant or epithelial cells (Beverley, 1980). Using this antibody to form rosettes 95–98% of marrow cells can be removed producing a smaller number of smears. We have compared the rosetting technique with density separation over lymphoprep previously described (Dearnaley *et al.*, 1981).

The standard method, hereafter referred to as "the density method", currently in use for preparation of marrow samples for EMA staining consists of removal of erythrocytes and mature granulocytes by centrifugation over Ficoll-Hypaque of density 1.007 (lymphoprep). Marrow samples were layered over lymphoprep and centrifuged at 400 *g* for 20 min. The cells retained at the interface were then aspirated, and pelleted. The pellet was resuspended in a small volume of sterile PBS and transferred to a siliconised 1 ml conical tip polypropylene tube. The cells were pelleted, transferred to the microscope slides and smeared. The smears were then immediately wet-fixed in 100% ethanol for 2 h.

Human erythrocytes (RBC) were coupled by chromium chloride to sheep anti-mouse Ig (SaM). The coupled erythrocytes (RBC.SaM) were added to the marrow samples previously incubated with mouse monoclonal anti-HLe-1. Rosettes formed around any cell bearing mouse Ig allowing density separation of the rosette-forming (i.e. normal marrow) cells. Chromium chloride was prepared by the "ageing" method (Goding, 1976). Briefly a 1% solution of chromium chloride ($CrCl_3$) in 0.9% sodium chloride (NaCl) was adjusted to pH 5 by the addition of 1 N sodium hydroxide twice-weekly for 3 weeks, then stored without further adjustment in a dark container. A 0.1% solution in 0.9% NaCl was prepared freshly from this stock just prior to use. Erythrocytes from fresh heparinised blood taken from healthy volunteers were washed 4× in 0.9% saline, and then pelleted. An aliquot of 85 $\mu$l of the packed RBC pellet was then resuspended in a suitable volume of 0.9% saline so that when the SaM and the $CrCl_3$ were added the resulting volume was 2 ml. The antibody used was a hetero-antiserum sheep-anti-mouse-immunoglobulin (SaM) dialysed extensively against 0.9% NaCl. An aliquot (0.5 mg) of this antibody was added and followed immediately by 135 $\mu$l of fresh 0.1% $CrCl_3$. After 5 min at room temperature with occasional agitation, the reaction was quenched with 3–5 ml of PBS, and red cells were washed twice in PBS and resuspended in 5 ml of medium. The red cells now coupled to SaM (RBC.SaM) were stored on ice overnight for use within 24 h.

The monoclonal antibody anti-HLe-1 was kindly provided as culture supernatant by Dr P.C.L. Beverley of the ICRF Tumour Immunology Unit, University College, London. This antibody recognises an antigen present to some degree on all lymphoid and myeloid cells in the marrow but not late normoblasts (Beverley, 1980). Studies using cell-lines and human tissues had shown no cross-reactivity with breast cancer or other epithelial tissues (Beverley, 1980). The supernatant was used at a final dilution of 1:50.

Correspondence: R. Buckman.

*Present address: Dept. Radiotherapy and Oncology, University College Hospital, Gower St, London WC1.

Received 4 July 1983; accepted 19 September 1983.

© The Macmillan Press Ltd., 1984

The rosetting method used was based on that of Parish & McKenzie (1978). The marrow sample, up to a total of $8 \times 10^7$ nucleated cells per universal container was incubated with anti-HLe-1 at 1:50 dilution for 1 h at room temperature and then washed twice in tissue culture medium (RPMI 1640, HEPES buffered, with 2% foetal calf serum). To each container 2 ml of the previously prepared RBC.SaM was added. In order to encourage rosette formation the marrow cells and RBC.SaM were compressed onto a Percoll cushion. It was found that this made resuspension of the formed rosettes easier and caused less loss of viability than using centrifugation at low gravity onto the bottom of the container. The cushion was formed by underlaying 2 ml of isotonic Percoll (prepared with 9 vol of neat Percoll to 1 vol of 10 x PBS) beneath the mixture using a long needle. The mixture was then centrifuged at 200 *g* for 5 min and then left at unit gravity for 10 min. The Percoll cushion was then carefully withdrawn from beneath the mixture which was then ready for separation.

Control experiments using leukocytes that had not been incubated with anti-HLe-1 showed that RBC.SaM did not form spontaneous rosettes around human leukocytes. Trypan blue preparations of rosetted marrow cells showed that rosettes were formed comprising 50–200 red cells per leukocyte.

In order to minimise non-specific loss of malignant cells into the pellet during separation of rosette-forming (marrow) cells, a higher density of Ficoll-Hypaque was used. Ficoll-Hypaque of density 1.100 was prepared by mixing 37 ml of lymphoprep with 15 ml of sodium diatrizoate (Hypaque). The density of random batches was checked with a 10 ml density bottle.

The marrow aspirate/RBC.SaM mixture, after removal of the Percoll cushion, was gently resuspended by inverting the tube 3 times. Then 10 ml of Ficoll-Hypaque of density 1.100 was underlayered. The samples were then centrifuged at 400 *g* for 20 min. The cells retained at the interface were then prepared for staining in the same way as those in the density method.

Immunocytochemical staining for EMA has already been described (Dearnaley *et al.*, 1981). Briefly, the endogenous alkaline phosphatase activity of osteoblasts was blocked by periodic acid, acetic acid and levamisole and the cells incubated with rabbit anti-EMA at 1:1000 dilution. After washing they were incubated with goat-anti-rabbit-immunoglobulin conjugated to alkaline phosphatase and the colour reaction developed by using Naphthol AS:Bl and Brentamine Fast Red TR to produce a red stain on all cells expressing EMA. Counterstaining was with Meyer's haemalum.

In order to find out whether the rosetting technique, in concentrating the marrow aspirates, removed significant numbers of malignant cells, two series of experiments were carried out: (i) Large volume single (10–15 ml) aspirates were taken from 20 consenting patients known to have bone involvement on bone scan or skeletal survey. The samples were divided into equal aliquots, one being prepared by the density method, the other by the rosetting method. (ii) In a further series of 46 patients with biopsy proven breast cancer, having a general anaesthetic for some other purpose (e.g. mastectomy, oophorectomy), marrow samples were aspirated from 8 sites (from bilateral anterior and posterior iliac spine from the upper and lower sternum and from 2 sacral sites). Theses samples were divided into equal aliquots in all but 9 experiments (where only one third of the sample was processed by the rosetting technique).

Table I shows that the rosetting technique removed an average of 97.3% and 94.2% of the marrow cells from the 26 single aspirates and 46 multiple aspirates respectively.

The density method produced an average of 5 smears in the single and 20 smears in the multiple aspirates series. The rosetting technique yielded an average of 2 smears in both series, representing a

**Table I** Efficiency of rosetting in both series of marrow aspirates

| | *Average volume of sample rosetted* | *No. of marrow cells at start* | *Marrow cells rosetted out (%)* | *No. of smears produced by density method* |
|---|---|---|---|---|
| Multiple aspirates (46 patients) | 7.1 ml | $6.8 \times 10^7$ ($3–19 \times 10^7$) | 94.2 (85–99.1) | 26 (18–41) |
| Single aspirate (20 patients) | 6.1 ml | $4.3 \times 10^7$ ($1.2–18.7 \times 10^7$) | 97.3 (84–99.9) | 5 (1–27) |

considerable reduction in the number of slides screened.

Table II compares the number of positive samples detected by the two methods. The rosette method produced a false negative result in 11 patients in whom malignant cells were detected by the density method and did not yield any positive samples when the density method was negative.

**Table II** Comparison of density method and rosette method results

| *Density method* | *Rosette method* | *No. of patients* |
|---|---|---|
| Positive | Positive | 17 |
| Negative | Negative | 38 |
| Positive | Negative | 11 |
| Negative | Positive | 0 |
| Total number compared | | 66 |

From Figure 1 it can be seen that the rosetting technique produced a negative result when there were only small numbers of malignant cells present (<6 cells). However, when larger numbers of cells were present both the rosetting and the density method yielded positive results.

Table III compares the results of the density method with the clinical status of the patients. It can be seen that 20% of patients with primary breast cancer with no evidence of metastases had demonstrable malignant cells in their marrow by analysis of multiple aspirates using the density method. Eighty percent of patients with known bony metastases in the single aspirate series and 75% of patients with other metastases in the multiple aspirate series had positive samples.

**Table III** Number of positive samples in series of multiple and single aspirates

| | *Multiple aspirate* (%) | *Large volume single aspirate* (%) |
|---|---|---|
| Primary breast cancer | 8/39 (20) | |
| Known evidence of bone metastases | 1/3 (33) | 16/20 (80) |
| Known evidence of other metastases (skin, lung, local) | 3/4 (75) | |

**Figure 1** Results of the rosetting technique in all samples in which malignant cells were detected by the density method.

The negative rosetting technique described here clearly offers a highly selective method of removing haematological cells from a cell suspension or marrow sample. Unfortunately, it may be of limited value in the early detection of metastatic spread of breast cancer to bone marrow since when 6 or less malignant cells are present in the sample, they are likely to be lost into the pellet instead of being retained at the interface.

The rosetting rechnique was devised in order to minimise the problem of non-specific nucleated cell loss, and the high density separation medium FH 1.100 is the highest density that will allow separation or erythrocytes so that further improvement or resolution of the problem is not likely with this technique.

From the clinical point of view, the significance of any malignant cells in the marrow can only be defined after long-term follow-up of these patients. Since there are no data to suggest any clinically significant "threshold" for malignant cells in the marrow, we felt that it would not be advisable to miss lower (i.e. <6) numbers of malignant cells. Therefore the rosetting technique is unsuitable for use on a routine basis for the clinical staging of patients with breast cancer.

However, there are other tumours in which it would be greatly desirable to detect early invasion of the bone marrow. This technique may be more suitable for tumours such as small cell carcinoma of the lung (SCCL) where the number of cells found in marrow is generally higher and where marrow involvement is more commonly detected by conventional staining. At the time of writing, there are no exceptionally useful monoclonal antibodies raised for the identifying metastatic cells from SCCL. However, workers at two centres are active in this field and both are planning to use the anti-HLe-1 rosetting technique to look for small numbers of malignant cells in patients with SCCL and apparently limited disease. Provided the cell numbers are larger than in breast cancer, the technique may be of value.

Negative rosetting may also have a role in the selective removal of leukocytes from cell mixtures. It has been used in this way to produce pure tumour cell populations from digests of metastatic lymph nodes and has enabled us to study cell surface antigens of pure human tumour cell populations.

### References

BEVERLEY, P.C.L. (1980). Production and use of monoclonal antibodies in transplantation immunology. In: *Transplantation and Clinical Immunology* XI (Ed. Touraine). Excerpta Medica: p. 87.

BRUNNING, R.D., BLOOMFIELD, C.D., MCKENNA, R.W. & PETERSON, L. (1975). Bilateral trephine bone marrow biopsies in lymphoma and other neoplastic diseases. *Ann. Int. Med.*, **82**, 365.

DEARNALEY, D.P., SLOANE, J.P., ORMEROD, M.G. & 7 others. (1981). Increased detection of mammary carcinoma cells in marrow smears using antisera to epithelial membrane antigen. *Br. J. Cancer*, **44**, 85.

GODING, J.W. (1976). The chronic chloride method of coupling antigens to erythrocytes: Definition of some important parameters. *J. Immunol. Method.*, **10**, 61.

HEYDERMAN, E., STEELE, K. & ORMEROD, M.G. (1979). A new antigen of the epithelial membrane: Its immunoperoxidase localisation in normal and neoplastic tissue. *J. Clin. Pathol.*, **32**, 35.

PARISH, C.R. & MCKENZIE, I.F.C. (1978). A sensitive rosetting method for detecting subpopulations of lymphocytes which react with alloantisera. *J. Immunol. Method*, **20**, 178.

SLOANE, J.P., ORMEROD, M.G., IMRIE, S.F. & COOMBES, R.C. (1980). The use of antisera to epithelial membrane antigen in detecting micrometastases in histological sections. *Br. J. Cancer*, **42**, 392.